# Specificity of the stabilizing interaction between intrinsically disordered protein sequences and G-quadruplexes in RNA

**DOI:** 10.1093/nar/gkaf1471

**Published:** 2026-01-06

**Authors:** Lachlan B Cox, John S Mattick, Isis A Middleton, Felix J Rizzuto, Pall Thordarson

**Affiliations:** School of Chemistry, University of New South Wales, Sydney, NSW 2052, Australia; UNSW RNA Institute, University of New South Wales, Sydney, NSW 2052, Australia; UNSW RNA Institute, University of New South Wales, Sydney, NSW 2052, Australia; School of Biotechnology and Biomolecular Sciences, University of New South Wales, Sydney, NSW 2052, Australia; School of Chemistry, University of New South Wales, Sydney, NSW 2052, Australia; UNSW RNA Institute, University of New South Wales, Sydney, NSW 2052, Australia; School of Chemistry, University of New South Wales, Sydney, NSW 2052, Australia; UNSW RNA Institute, University of New South Wales, Sydney, NSW 2052, Australia; School of Chemistry, University of New South Wales, Sydney, NSW 2052, Australia; UNSW RNA Institute, University of New South Wales, Sydney, NSW 2052, Australia

## Abstract

Intrinsically disordered regions (IDRs) are present in and essential for the function of nearly all the proteins involved in regulation, cell, and developmental processes. The RGG domain in IDRs binds ‘promiscuously’ to RNA G-quadruplexes (rG4s), a non-canonical 4-stranded secondary structure that occurs in many transcripts involved in gene regulation. Here we show, using weak binding interactions between a minimal RGG-rich peptide and rG4s, that the IDR selectively templates and stabilizes the structure of the human telomeric TERRA rG4, providing a unique pathway to RNA folding that does not rely on high-affinity binding or monovalent cations. Multidimensional NMR and circular dichroism (CD) spectroscopy analyses reveal individual nucleotide and amino acid identities determine the specificity of the interaction between RGG peptides and rG4s, explaining how IDRs can selectively recognize RNA over DNA G4s, the high specificity of such interactions *in vivo*, and the high frequency of monogenic mutations observed in IDRs.

## Graphical Abstract

**Graphical Abstract:**
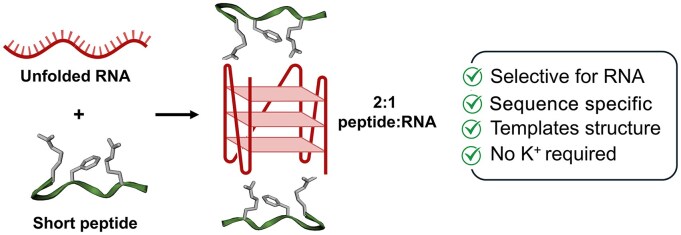

## Introduction

**Figure 1.**
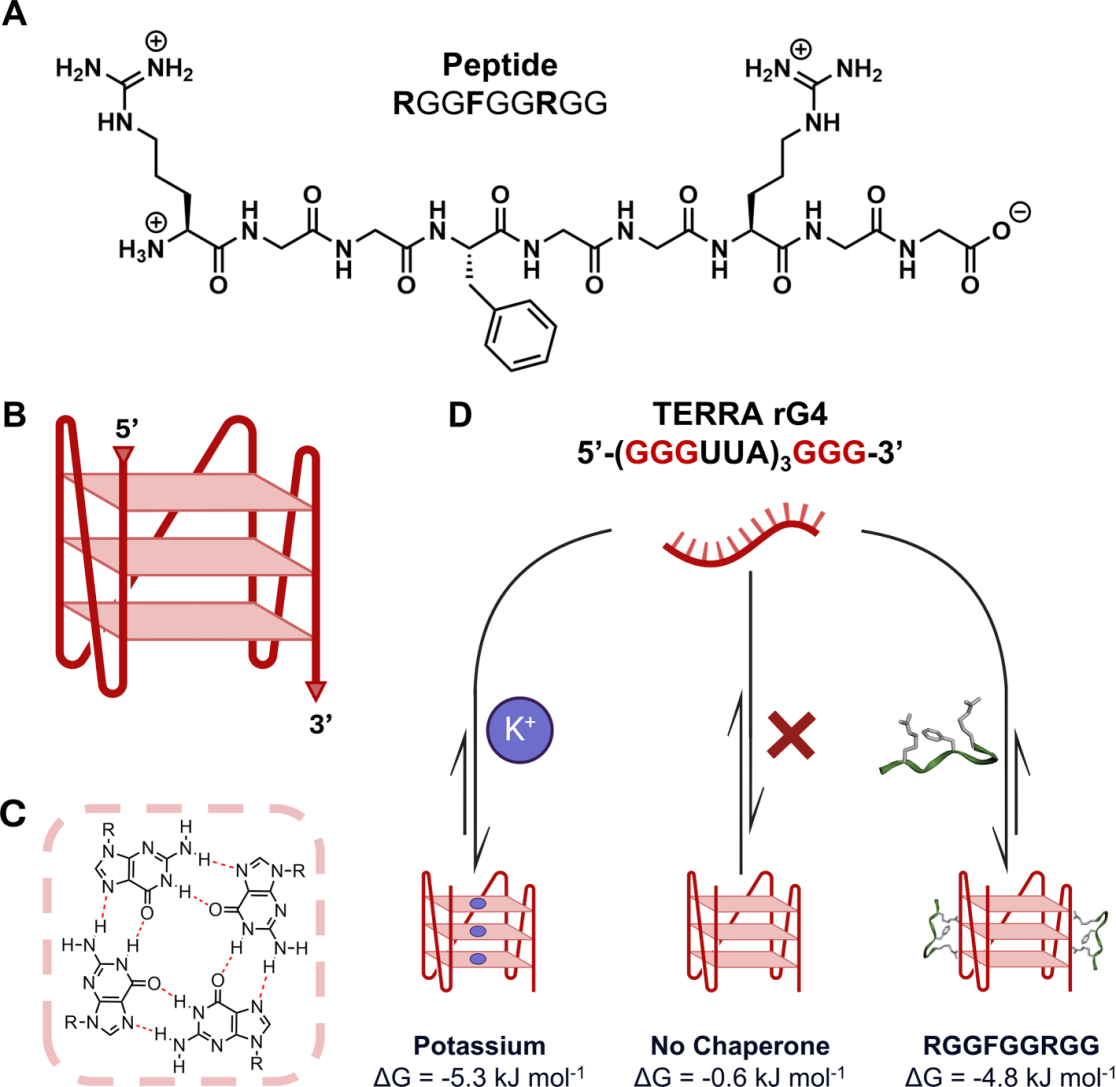
Peptide and oligonucleotide sequences used in this study. (**A**) Sequence of the RNA-binding peptide RGGFGGRGG, derived from the intrinsically disordered region of nucleolin. (**B**) Folded structure of the TERRA rG4 structure. (**C**) Four guanines can interact via Hoogsteen hydrogen bonding to form a tetrad sheet. (**D**) The rG4 structure can be chaperoned by either potassium ions (left) or the RGGFGGRGG peptide (right) to a comparable magnitude. The free energy of templation (ΔG) at 25°C is shown below each structure. The 5′ to 3′ RNA phosphate backbone is represented by thick lines and the Hoogsteen base pairs are indicated by thin horizontal sheets. Created in BioRender. Cox, L. (2025) https://BioRender.com/h6hjuuq.

Nucleic acids form diverse secondary structures, many of which modulate cellular functions [1]. Apart from the canonical Watson–Crick duplex, DNA and RNA can form non-canonical structures under specific conditions [2–4]. Among these, G-quadruplexes (G4s) are the most prevalent folded non-canonical structures in the genome [5]. These motifs are thought to be involved in genetic disorders by altering transcriptional, translational, and epigenetic regulatory factors [6], resulting in neurodegenerative diseases [7], myotonic dystrophy, and cancers [8]. G4s are formed of planar guanine tetrads, held together by self-complementary hydrogen bonding templated around a central monovalent cation (Fig. 1C) [9], and occur in a wide variety of messenger RNAs and long noncoding RNAs (lncRNAs), including those involved in telomere maintenance, enhancer action, modulation of chromatin structure, transcription, RNA splicing, RNA transport, and translation [10–12]. The termini of human chromosomes—the telomere—contain instructions for this structure in cells, with capping regions of 300–8000 tandem repeats of 5′-CCCTAA/TTAGGG-3′ [13]. This region is tasked with regulating telomere integrity upon interaction with proteins [14]. When transcribed, the telomeric repeat-containing, non-coding RNA (TERRA) sequence (GGG UUA)_n_ is formed, folding into a parallel G4 structure [15].

G4s interact strongly with the intrinsically disordered regions (IDRs) of proteins [16], long chains of amino acids that lack rigid tertiary structure and are characterized by a high proportion of small, polar, and positively charged amino acids (glycine, arginine, histidine, and lysine), often in the form of RGG/RG, histidine-rich domains, or other repeats [17–20]. IDRs are essential for the function of nearly all of the proteins involved in gene regulation, including RNA polymerase, most transcription factors, enhancers, Hox proteins, histones, histone-modifying proteins, other chromatin-binding proteins, the Mediator complex, RNA-binding proteins, splicing factors, membrane receptors, cytoskeletal proteins, and nuclear hormone receptors [21–35]. IDRs are also major sites of post-translational modifications and interact with a multitude of RNAs in the formation of biomolecular condensates, or ‘phase-separated domains’, in the nucleus and cytoplasm [36–38].

Like G4 quadruplexes, IDRs are described as ‘promiscuous’, i.e. they interact with many partners [19, 20, 26, 39–51]. The aberrant promiscuity of IDR-containing proteins and perturbations of phase-separated domains may underlie the dosage sensitivity of oncogenes and other proteins [52, 53] as well as neurodegenerative disorders [54].

One of the most common RNA-binding motifs in IDRs is the RGG domain, which binds to G-quadruplexes in RNAs (rG4s), reciprocally mediating degenerate specificity [55]. The specificity of the RGG-rG4 interaction is poorly understood. A previous study established that multiple hydrogen bonds are involved in the interaction of an RGG peptide from the human fragile X mental retardation protein (FMRP) and an *in vitro*–selected guanine-rich RNA, validated by RNA and peptide mutations [56]. The IDR-directed binding of two different transcription factors *in vivo* was also shown to be due to weak determinants distributed throughout the IDR sequence [47], and phenylalanines adjacent to RGG motifs in the IDR of nucleolin are responsible for rG4 binding and folding [57].

Despite the importance of IDR–RNA interactions in biological systems, no studies have thus far confirmed the role of specific intermolecular interactions in stabilizing, forming, or disrupting non-canonical RNA secondary structures with proteins rich in IDRs [12, 57, 58]. To gain a holistic understanding of the nature and specificity of RNA–protein interactions in the formation of biocondensates [59] and the perturbation of these interactions in disease, unravelling the relatively weak interactions contributing to IDR–RNA binding is key.

Here we analyse in detail the interactions between the peptide RGGFGGRGG, derived from the IDR of the nucleolin RNA binding protein (Fig. 1A), and folded G4 structures from the human telomeric repeat-containing lncRNA (TERRA) sequence [5′-(GGG UUA GGG UUA GGG UUA GGG-3′] (Fig. 1B) [15, 60]. We show that this peptide stabilizes short natural sequences of human telomeric RNA to form an rG4 secondary structure, despite a relatively modest binding affinity. The peptide discriminates binding RNAs at a single-nucleotide mutation, is selective for RNA over DNA, and is competitive with the endogenous chaperone K^+^ (Fig. 1D). Mutation of the peptide sequence abrogates or severely weakens its binding, highlighting the essential amino acid residues of arginine and phenylalanine required for RNA stabilization. Our results are consistent with the observation that mutations that cause human monogenic diseases occur more commonly in IDRs than globular domains [61, 62], indicating that, despite their relatively simple composition, IDRs have strong sequence constraints.

## Materials and methods

### Solid-phase peptide synthesis and purification

The peptides used in this study were synthesized through standard solid-phase peptide synthesis (Supplementary Section 2.2). The purification was performed on a Shimadzu Prominence UFLC HPLC fitted with a Shimadzu 20AB pump and an SPD-20A photodiode array detector. An Intersil ODS-4 5 μm 20 × 150 mm column with a C18 stationary phase was used with mobile phases A: MilliQ™ water with formic acid (0.1%, v/v), and B: acetonitrile with formic acid (0.1%, v/v). The concentration of mobile phase B increased from 10% to 95% over 35 min, with a flow rate of 5 ml min^−1^ and monitored at 254 nm. Samples were lyophilized to afford purified peptide as a white powder.

### Solid-phase oligonucleotide synthesis and purification

RNA oligonucleotides were synthesized by the UNSW RNA Institute on an ÄKTA Oligopilot Plus 10 synthesizer at a 50 μmol scale. Phosphoramidites (Thermo Fisher Scientific) were dissolved to 0.1 M in anhydrous acetonitrile (Merck). All reagents were used as supplied without further purification.

Oligonucleotides were cleaved and deprotected from the solid support in a mixture of aqueous ammonia (40%, 2 ml) and methylamine (33%, 2 ml) (55°C, 50 min). Secondary deprotection of TBDMS groups was achieved with TEA·3HF (1 ml) in dimethyl sulfoxide (1 ml) (65°C, 3 h), followed by precipitation and washing in ice-cold butanol.

Purification was performed by strong anion exchange HPLC (SAX-HPLC) using a ThermoScientific™ DNAPac™ PA 100 BioLC™ column (22 × 250 mm) on a Shimadzu LC-20AP system with a Shimadzu 20AB pump and an SPD-M40 photodiode array detector with mobile phases: A: Milli-Q™ water with sodium hydroxide (5 mM, pH 12), and B: Milli-Q™ water with sodium chloride (1 M) with sodium hydroxide (5 mM, pH 12). The concentration of mobile phase B was increased from 20% to 80% to 95% over 60 min with a flow rate of 5 ml min^−1^ and monitored at 260 nm. Desired fractions were collected, neutralized with dilute HCl, lyophilized, and desalted using Amicon Ultra 3k centrifugal filters.

### Secondary structure assembly and buffer conditions

RNA secondary structures were annealed in the desired buffer using an Applied Biosystems ProFlex PCR system. Oligonucleotides were heated at 85°C for 15 min, followed by cooling at 0.1°C/min to 20°C, unless otherwise stated. Experiments were performed in 50 mM potassium phosphate buffers, with added KCl (50 mM) unless otherwise stated, pH adjusted to 7.00 using HCl/KOH. Unless otherwise stated, experiments were performed at 25°C. The pH of the buffers and samples was measured with a Hanna Instruments HI11310 pH probe.

### Ultraviolet-visible and circular dichroism spectroscopy

Oligonucleotide purity and concentration were determined using a Thermo Fisher NanoDrop One/OneC microvolume UV spectrophotometer. One microlitre of sample in Milli-Q™ water measured at (A260/A280 ≥ 2.0). Molar extinction coefficients were calculated with the IDR OligoAnalyzer™ tool.

Circular dichroism (CD) and UV-Vis spectra were recorded in a 1 mm quartz cuvette on an Applied Photophysics Chirascan Plus CD Spectrometer. Samples (20 μM) were dissolved in potassium phosphate (50 mM) and potassium chloride (50 mM) at pH 7.01. CD spectra were obtained at 25°C with an interval and bandwidth of 1 nm.

For melting curve titrations, RNA samples (20 μM) were prepared in 1× tris acetate buffer at pH 7.03 and titrated with 1–16 equivalents of RGGFGGRGG. Ellipticity at 260 nm (parallel) or 295 nm (antiparallel) [63] was monitored every 0.2°C with a temperature gradient of 0.6°C min^−1^ from 5°C to 95°C. Data were fit to an asymmetric sigmoidal nonlinear regression to determine the melting temperature.

### Nuclear magnetic resonance spectroscopy

^1^H nuclear magnetic resonance (NMR) spectra were obtained on a Bruker Avance III 600 MHz Cryo NMR spectrometer. Peptide and oligonucleotide samples were prepared in 10% D_2_O : 90% buffer. Water suppression was achieved using excitation sculpting. Unless otherwise stated, spectra were collected at 25°C.

Secondary structure and binding mechanism were determined by multidimensional NMR spectroscopy (HSQC, TOCSY, and NOESY). Peptide:RNA proximity crosspeaks observed by NOESY were validated by saturation transfer difference (STD) NMR spectroscopy. Oligonucleotide samples were prepared to a concentration of 2 mM in 10% D_2_O : 90% buffer.

### NMR titrations

NMR titrations were performed using a constant concentration of peptide (125 μM) and titrated with 0–4 equivalents of oligonucleotide. Samples (160 μl) were prepared in 3 mm NMR tubes in 10% D_2_O : 90% buffer. A minimal concentration of 3-(trimethylsilyl)propinoic-2,2,3,3-*d*_4_ acid sodium salt was included as an internal standard. A discussion of the models and data fitting is available in Supplementary Information 1.3.

## Results

### Rational design of short RNA-binding peptides derived from nucleolin

The human nucleolin RNA-binding protein is a multifunctional transcription regulator involved in ribosomal biogenesis, RNA metabolism, and chromatin formation [64]. Nucleolin is known to interact with G-rich RNAs *in vivo* [58], with recent studies demonstrating its stabilizing effect on RNA G-quadruplex (rG4) structures [57]. Nucleolin facilitates rG4 binding through its C-terminal RG/RGG-rich IDR [55, 65].

The structural complexity and vast size of biomacromolecules make it challenging to study specific intermolecular interactions between proteins and nucleic acids, especially for weakly associating RNAs and peptides. Using small-scale synthetic replicas of biological systems allows the probing of specific peptide-oligonucleotide interactions on a molecular level, which may lead to the future design of peptide targeting agents for therapies [66], drug delivery [67], and artificial chemical systems [68]. It is estimated that up to 97% of the RNA produced in cells is non-protein-coding [69], many of which are involved in the formation of biomolecular condensates in the nucleus and cytoplasm [70, 71], including the action of genetic loci called ‘enhancers’, which control the spatiotemporal patterns of development [72, 73]. Unravelling the range of weak interactions of these non-coding RNAs with IDRs is key to understanding their functional roles in cellular and developmental regulation.

To elucidate the binding dynamics of IDR peptides with rG4s, three short, rationally designed peptide sequences—1: RGGFGGRGG (R644-G652) (Fig. 1A), 2: RGGRGGRGG, and 3: LGKGGYGKV—were derived from the Nucleolin protein. The arginine residues in peptides 1 and 2 promote affinity for the RNA phosphate backbone through electrostatic attraction and guanidinium-phosphate hydrogen bonding [74]. The phenylalanine residue in peptide 1 allows for π–π stacking with the aromatic nucleobases, which is thought to be essential for nucleolin’s interactions *in vivo* [58, 75]. Glycine residues provide structural flexibility to the peptide backbone, allowing the peptide to conform to the RNA grooves for optimal binding [12]. Peptide 3 was designed as a negative control; although it retains a 2^+^ charge, aromatic residue, and glycine flexibility, it lacks the characteristic RGG repeat motif.

**Figure 2.**
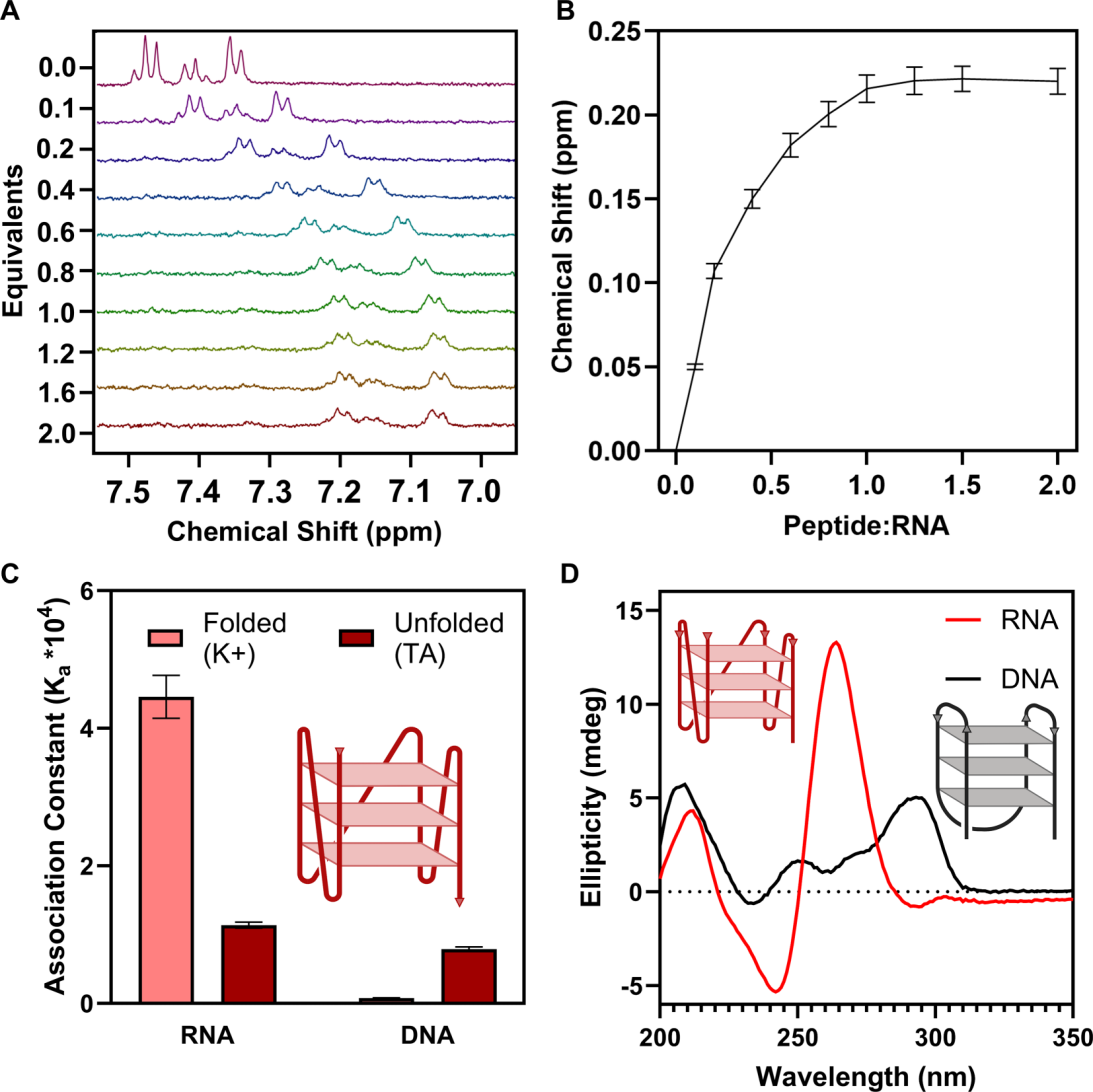
The RGGFGGRGG peptide is selective for TERRA rG4s over a DNA counterpart and discriminates for folded structures. (**A**) ^1^H NMR titration data of the RGGFGGRGG peptide titrated with TERRA RNA (0–2 equivalents). Region showed corresponds to phenylalanine aromatic protons. (**B**) Binding isotherm of the RGGFGGRGG aromatic phenylalanine protons upon being titrated with TERRA RNA. (**C**) 2:1 statistical association constants determined by supramolecular.org between the peptide and G4-forming 5′-(GGGUUA)_3_GGG-3′ sequences: RNA versus DNA (U → T) in the folded (K^+^) or unfolded (TA) states. (**D**) CD spectra of TERRA RNA and DNA G4 structures. RNA forms a parallel rG4, while DNA forms a predominantly antiparallel G4, as indicated by the arrows on the schematic 5′ to 3′ backbones. Created in BioRender. Cox, L. (2025) https://BioRender.com/7oljjqq.

### RGGFGGRGG selectively binds the TERRA rG4 over the DNA counterpart

To evaluate whether RGGFGGRGG binds preferentially to folded RNA G-quadruplexes (rG4s), we determined the association constants between RGGFGGRGG and TERRA rG4 using ¹H NMR titrations performed in duplicate. NMR titrations were performed with a constant concentration of RGGFGGRGG (125 μM) and increasing ratios of RNA or DNA, from 0 to 2 equivalents. The change in chemical shift (Δδ) of the aromatic phenylalanine protons of the RGGFGGRGG peptide was monitored (Fig. 2A), converted to binding isotherms (Fig. 2B), and analysed using the online tool supramolecular.org (76, http://supramolecular.org), with the weighted average association constants (M^−1^) reported (Fig. 2C).

^1^H NMR titrations using the RGGFGGRGG peptide showed that the TERRA rG4 sequence—5′-GGG UUA GGG UUA GGG UUA GGG-3′—interacted with a ~4-fold higher association constant when folded in a potassium-containing buffer, as compared to the same RNA sequence that was unfolded in the absence of potassium (ΔΔG = –3.2 kJ mol^−1^, Fig. 2C). The peptide further exhibited a ~50-fold selectivity in terms of association constants for the stepwise 2:1 peptide-to-RNA equilibria for the folded rG4 over the DNA variant—5′-GGG TTA GGG TTA GGG TTA GGG-3′—(ΔΔG = –10.0 kJ mol^−1^). Isothermal Titration Calorimetry results agreed with the binding constants obtained from NMR titrations (Supplementary Fig. S8).

We compared fitting models for the folded and unfolded states of TERRA RNA and DNA interacting with RGGFGGRGG (Supplementary Tables S2 and S3). In all cases, the stoichiometry was best described as two peptide molecules interacting with one nucleic acid structure. The most appropriate model for the majority of titrations was a non-cooperative statistical 2:1 host-guest model [77], which is the simplest model with the fewest forced parameters [76]. This fitting model suggests that two peptide molecules bind to the TERRA rG4 independently and that the binding of the two peptides to each nucleic acid strand is a purely statistical 2:1 process with negligible cooperativity, implying a symmetrical binding event [76, 77].

We propose that the observed selectivity for RGGFGGRGG to RNA arises in part from the difference in folding conformation between the RNA and DNA structures. RNA exclusively adopts a parallel rG4 structure, characterized using CD spectroscopy by the strong positive band at 262 nm and large negative band at 245 nm [78]. In contrast, the DNA G4 adopts an antiparallel topology, characterized by a positive band at 295 nm and a negative trough at 232 nm (Fig. 2D) [63]. These folding conformations place the tetrad sheet at a different spatial configuration, as well as orienting the loop nucleotides differently: RNA bulges to the side to form a shorter, wider ‘sandwich’ structure, while DNA protrudes at the top and bottom to create a taller, thinner ‘chair’ structure [79]. We theorize that the antiparallel G4 structures may not present an ideal binding pocket on the external backbone for the peptide to interact strongly, resulting in a decreased association constant as compared to parallel G4s.

### RGGFGGRGG templates and thermodynamically stabilizes the TERRA rG4

To assess the impact of the RGGFGGRGG peptide on the stability of the TERRA rG4 structure, we conducted CD melting-curve titrations [9] in the absence of potassium. Hoogsteen structures—including G4s—are generally less stable than Watson–Crick helices under physiological conditions (80) and typically require precise folding conditions, such as the presence of monovalent cations for G4s. For example, rG4 structures often denature upon single-point mutations, leading to a loss of function and RNA misfolding disorders [55]. Selective nucleic acid binders that can influence the stability of these structures are therefore of great interest [81]; however, protein-inspired peptides have not featured heavily in terms of selectivity for Hoogsteen-based nucleic acid structures [68, 82].

In our CD melting point assays, ellipticity at 260 nm (the characteristic wavelength of parallel G4s [63]) was monitored across a temperature gradient from 5°C to 95°C. The melting temperature (T_m_) was defined as the temperature where 50% of the structure had denatured. A baseline CD spectrum of TERRA RNA folded into an rG4 by 100 mM K^+^ at 5°C was used as the ‘100% folded’ reference. All subsequent measurements were normalized to this value to determine the percentage of folded rG4 structure upon the addition of RGGFGGRGG. Melting point experiments on the ‘unfolded’ TERRA and 16 equivalents of peptide were performed in triplicate.

**Figure 3.**
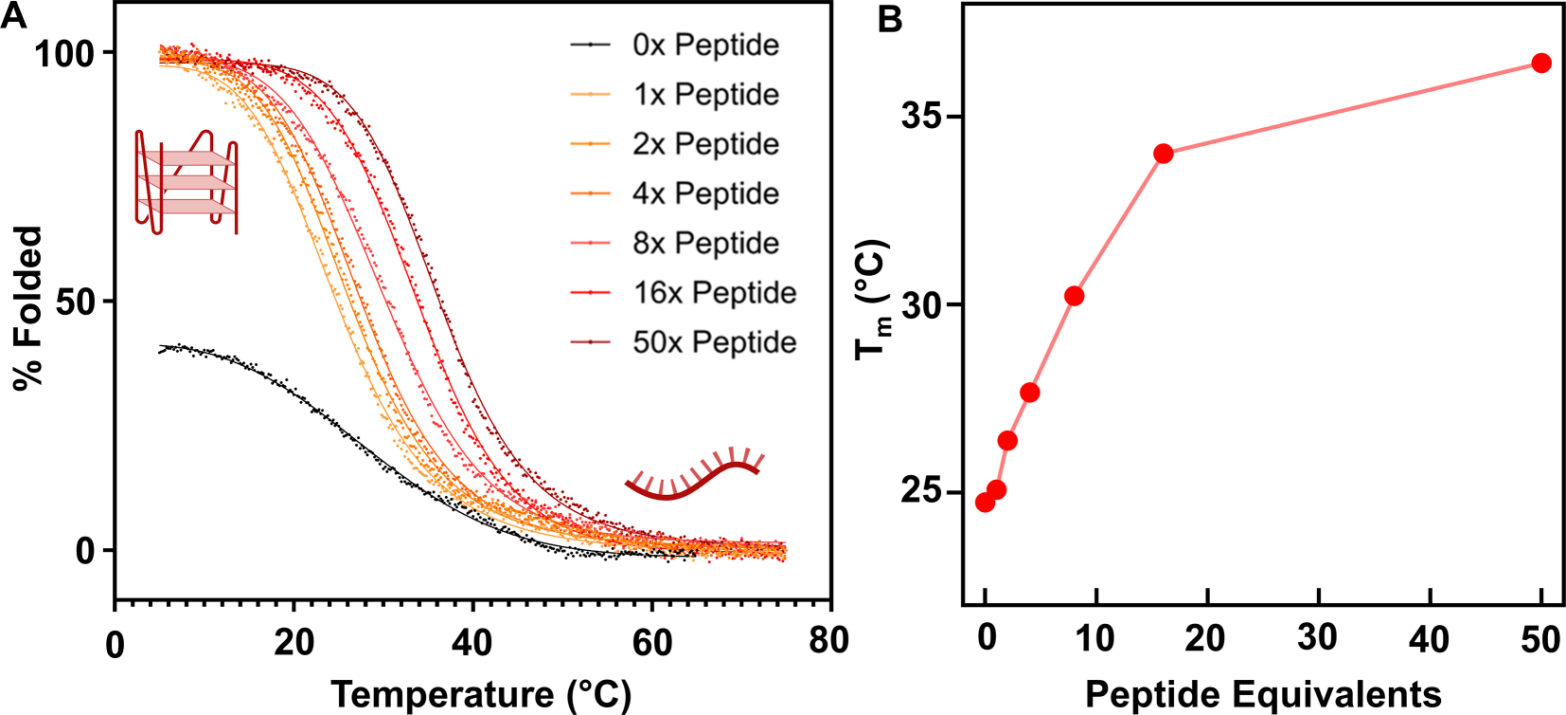
Additions of RGGFGGRGG selectively increase the thermal stability of the TERRA rG4 in the absence of potassium. (**A**) CD melting curves of the TERRA rG4 upon increasing equivalents of RGGFGGRGG (20 μM RNA, 1 × tris acetate buffer at pH 7.0). Solid line is a non-linear regression best fit to the data. (**B**) Melting temperatures (T_m_) of the TERRA rG4 at each tested peptide equivalent, interpolated from the melting curves in panel (A). Created in BioRender. Cox, L. (2025) https://BioRender.com/k62in3j.

In the absence of salt or peptide, the melting temperature (T_m_) of the TERRA rG4 was 24.7 ± 0.1°C. Although this measurement represents the ‘unfolded’ control, a residual CD peak at 260 nm corresponding to ∼40% folded structure was observed, attributed to the inherent A-form helix of RNA [63] and trace sodium counterions from synthesis, which can weakly template G4 structures [63]. Upon the addition of one equivalent of the RGGFGGRGG peptide, the proportion of folded rG4 increased to nearly 100%. UV-visible spectroscopy confirmed that the peptide folds the structure to a comparable extent as the endogenous ligand K^+^, as shown by comparable bathochromic and hypochromic shifts (Supplementary Fig. S9). Further titration of RGGFGGRGG into the TERRA RNA resulted in a stepwise increase in the melting temperature of the rG4 structure. Upon the addition of 16 equivalents of peptide, the T_m_ increased to 34.0 ± 0.1°C (ΔT_m_ = 9.3°C, Fig. 3B).

The stabilization tells us that not only does RGGFGGRGG bind to pre-folded TERRA rG4s, but it can also act as a ‘molecular chaperone’ for the TERRA rG4, promoting structure formation and increasing the thermodynamic stability even in the absence of monovalent cations. This behaviour mimics the role of cellular RNA-binding proteins that are likely essential for lncRNA structure and function *in vivo*, showing that minimalist IDR-derived peptides can replicate the structural roles of full-length proteins.

### RGGFGGRGG weakly stabilizes but does not template the parallel pU27 DNA G4

RNA exclusively adopts a parallel G4 structure—no examples of antiparallel rG4s exist without modified or unnatural bases [83, 84]. Conversely, DNA adopts a hybrid conformation, heavily favouring antiparallel; however, there are a few examples of parallel DNA G4s [85]. To assess if the stabilization of the TERRA rG4 by RGGFGGRGG arises primarily due to the parallel folding topology of RNA rather than sequence specificity, we conducted additional CD melting curves with a parallel DNA G4, the pU27 sequence—5′-TGG GGA GGG TGG GGA GGG TGG GGA AGG-3′. pU27 is a G-rich tract of DNA found in the Nuclease Hypersensitive Element III1 (NHE III1) region of the c-MYC P1 promoter [86], and its parallel G4 structure has been linked to transcriptional repression of aberrant c-MYC expression, resulting in suppression of cancer proliferation [87]. Studies have shown that the nucleolin protein (the source of the RGGFGGRGG peptide) binds and stabilizes the pU27 G4 *in vivo* [58].

**Figure 4.**
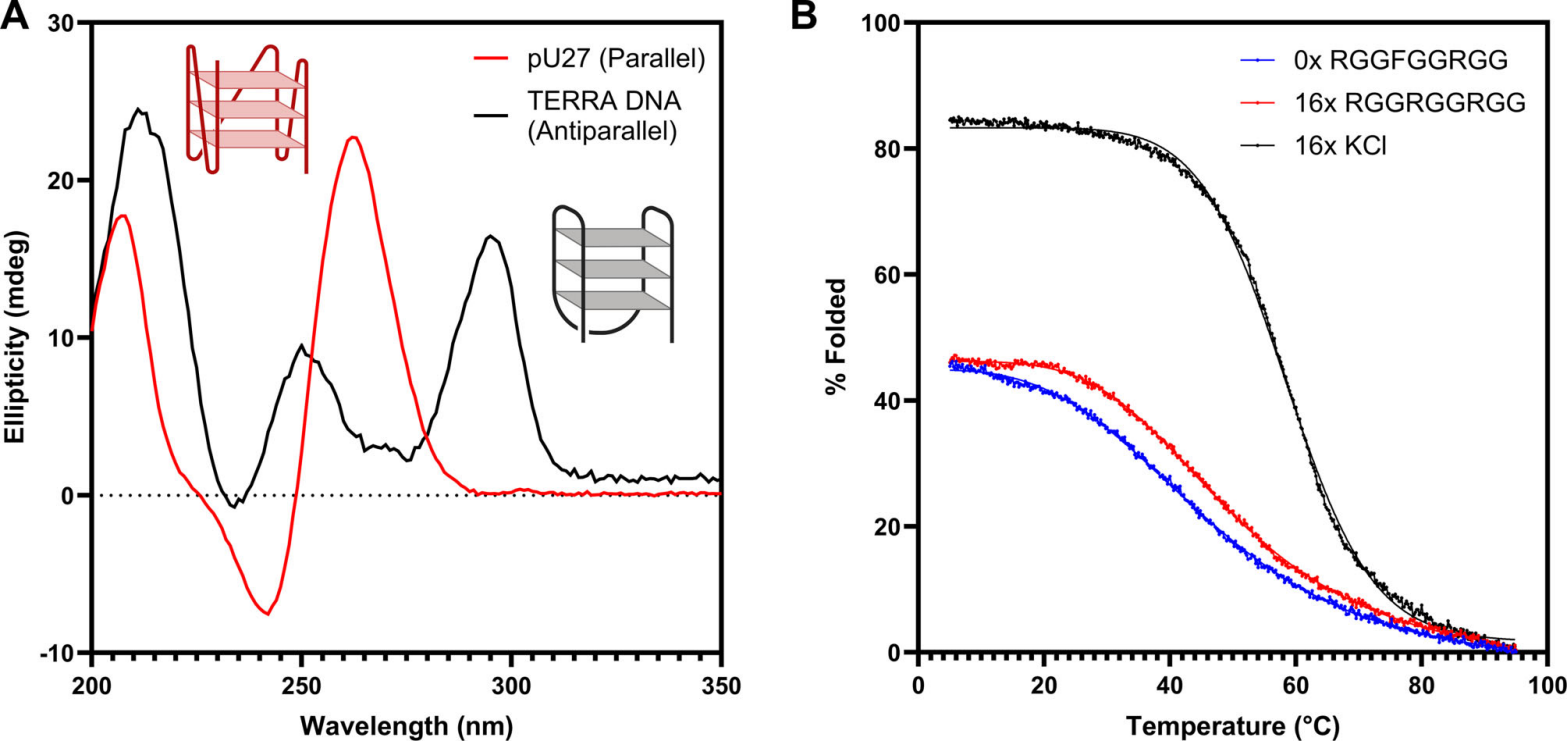
The RGGFGGRGG peptide increases the thermal stability of the parallel pU27 DNA G4 but cannot template its formation. (**A**) CD spectra of the parallel pU27 sequence (red), as compared to the antiparallel TERRA DNA (black) in the presence of potassium (20 μM RNA, 50 mM KCl, 50 mM potassium phosphate pH 7). (**B**) Circular dichroism melting curves of the pU27 parallel G4 structure (blue) upon 16 equivalent additions of the RGGFGGRGG peptide (red) and the endogenous ligand potassium (black) (20 μM RNA, 1 × tris acetate buffer at pH 7.0). Solid line is a non-linear regression best fit to the data. The RGGFGGRGG peptide moderately stabilizes the pU27 G4 (ΔT_m_ = 5.0°C), whereas the endogenous ligand potassium fully templates the structure and stabilizes it to a higher degree (ΔT_m_ = 12.9°C). Created in BioRender. Cox, L. (2025) https://BioRender.com/kg5apd1.

We first used CD spectroscopy to confirm that the pU27 sequence formed a parallel G4 in the presence of potassium (Fig. 4A), in contrast to the antiparallel G4 formed by the DNA variant of TERRA. CD melting curves showed in the absence of potassium, the pU27 DNA sequence formed a partially folded parallel G4 structure (∼45% folded, T_m_ = 45.7°C). Upon the addition of 16 equivalents of RGGFGGRGG, the melting point increased to 50.7°C (ΔT_m_ = 5.0°C); however, we did not observe an increase in proportion of folded G4, suggesting that RGGFGGRGG modestly stabilizes but does not template the pU27 G4 structure (Fig. 4B). Upon the addition of 16 equivalents of the endogenous ligand K^+^, the melting point further increased to 58.6°C (ΔT_m_ = 12.9°C), and the parallel G4 structure was templated (Fig. 4). Sixteen equivalents of peptide were chosen as binding simulations using supramolecular.org predicted a 90% saturation at 16 equivalents of the RGGFGGRGG peptide to TERRA RNA in the absence of potassium (http://supramolecular.org).

To probe structural and sequence selectivity, we tested RGGFGGRGG against two additional DNA G4 sequences, the antiparallel DNA variant of the TERRA sequence—5′-GGG TTA GGG TTA GGG TTA GGG-3′—and an additional parallel DNA G4 sequence containing only adenines in the loop—5′-GGG AA GGG AA GGG AA GGG-3′. Neither sequence was stabilized or templated by the RGGFGGRGG peptide (Supplementary Fig. S10).

This series of CD melting curves suggests that the RGGFGGRGG peptide selectively stabilizes the pU27 parallel DNA but cannot act as a ‘molecular chaperone’, in contrast to its chaperoning effect on the TERRA rG4. This specificity may be rationalized by the comparatively lower hydration of the DNA G4 minor groove in comparison to RNA, which may limit hydrogen bonding with the hydrophilic peptide [88], preventing it from acting as a chaperone. Additionally, the observed increase in thermodynamic stability of the parallel pU27 G4, but not for the antiparallel TERRA DNA G4, suggests that RGGFGGRGG interacts preferentially with parallel over antiparallel G4 structures. As RGGFGGRGG increases the thermal stability of the parallel pU27 sequence but not the parallel (G_3_A_2_)_n_ sequence, we propose that the interaction depends not only on structure, but also displays sequence selectivity involving loop base identity (in this case, thymine > adenine).

### Sequence selectivity of RGGFGGRGG towards TERRA rG4

The RGGFGGRGG peptide both stabilizes and templates the TERRA rG4. To probe its sequence selectivity for the native TERRA structure and to determine key nucleobases required for binding, we performed additional CD melting curves on mutant TERRA sequences. From our previous results with pU27, we determined that specific loop nucleotides are significant for recruiting peptide binding and thus stabilization. Following this observation, we mutated the native TERRA loop sequence—UUA—to UUU and AAA, and then sequentially shortened it to two and one nucleotide (U_3_, U_2_, U_1_, A_3_, A_2_, A_1_). This approach not only demonstrates the impact of point mutations on peptide-mediated TERRA rG4 stabilization, but also reflects the diversity of rG4 sequences found across lncRNAs. For example, a tandem repeat of (GGGA_1-3_)_n_ is representative of c-MYC-associated transcripts [89, 90], while (GGGU_1-3_)_n_ tandem repeats are broadly prevalent in the human genome [91]. CD spectroscopy confirmed that all tested RNA sequences form rG4s (Supplementary Fig. S11A–F).

Upon mutation of the native TERRA tandem repeat (GGG UUA)_n_ to a loop containing only uracil (G_3_U_3_)_n_, there was a reduction in the observed stabilization in melting point (ΔT_m_) to ~4°C. Although there was still minor stabilization observed, the RGGFGGRGG peptide no longer induced complete formation of the rG4 structure, and the extent to which the G4 structure was folded at 25°C was no longer at a comparable magnitude to potassium (Table 1 and Supplementary Fig. S12A–F). Reductions in the length of the uracil loop progressively decreased stabilization (Table 1). When the loop sequence was instead mutated to only contain adenine (G_3_A_3-1_)_n_, RGGFGGRGG lost all stabilizing ability, and instead a slight destabilization of the rG4 was observed (ΔT_m_ = –2°C). In contrast, additions of the endogenous ligand K^+^ increased the stability of all tested mutant sequences and induced formation of the rG4 structure (Table 1 and Supplementary Fig. S12A–F).

**Table 1.** RGGFGGRGG selectively stabilizes the native TERRA rG4 structure over loop mutation sequences, whereas the endogenous ligand K^+^ stabilizes all rG4 structures

	Control (RNA control)	RGGFGGRGG (16×)		KCl (16×)	
Sequence (5′–3′)	T_m_ (°C)	T_m_ (°C)	ΔT (°C)	T_m_ (°C)	ΔT (°C)
(GGGUUA)_n_ (TERRA)	24.7 ± 0.1°C	34.0 ± 0.1°C	9.3	46.3 ± 0.2°C	21.5
(G_3_U_3_)_n_	31.3 ± 0.3°C	35.4 ± 0.2°C	4.1	45.3 ± 0.2°C	14.0
(G_3_U_2_)_n_	36.0 ± 0.1°C	40.9 ± 0.2°C	4.8	54.4 ± 0.1°C	18.4
(G_3_U_1_)_n_	50.9 ± 0.2°C	52.5 ± 0.2°C	2.4	61.8 ± 0.2°C	10.9
(G_3_A_3_)_n_	43.9 ± 0.2°C	41.5 ± 0.1°C	−2.4	58.7 ± 0.6°C	14.9
(G_3_A_2_)_n_	42.7 ± 0.2°C	36.9 ± 1.4°C	−5.8	45.3 ± 0.2°C	2.6
(G_3_A_1_)_n_	52.7 ± 0.6°C	52.5 ± 0.1°C	−0.3	59.6 ± 0.2°C	6.9

Melting temperatures (T_m_) were determined by CD melting curves, with uncertainty derived from nonlinear fitting error. The change in melting temperature (ΔT) is given relative to the control in the absence of monovalent cations or peptide. Each sample was measured with either 16 equivalents of RGGFGGRGG or the endogenous ligand, K^+^.

These results suggest that uracil is an essential loop residue for recruiting peptide binding, leading to stabilization of the TERRA rG4 structure, whereas adenine loops are unfavourable for RGGFGGRGG interactions, mimicking the conclusions of the pU27 screens, where thymine was preferable over adenine. As specific G4 loop base identity is significant in recruiting peptide interaction, it is evident that electrostatics alone are not responsible for these RNA-peptide binding interactions [92]. Notably, no tested permutation of loop sequence was stabilized by the peptide to the extent of the native TERRA sequence, highlighting the significance of evolved sequence selectivity of the RGGFGGRGG peptide towards the native TERRA rG4.

### Phenylalanine is essential for peptide-mediated TERRA rG4 stabilization

Having established that the parallel TERRA rG4 structure and uracil loop nucleotides are essential for RGGFGGRGG interaction and stabilization, we next mutated the peptide sequence to determine the contribution of individual amino acid residues. Biological studies have shown that nucleolin can stabilize large tracts of guanine-rich DNA. Upon mutation of nucleolin phenylalanine residues, there was a loss of stabilization [58]. To investigate the importance of phenylalanine in our system, we introduced a point mutation (4F→R) to afford the mutated peptide RGGRGGRGG. This mutation removes the ability of the peptide to π–π stack with the guanine tetrad of the rG4 structure while retaining its electrostatic attraction to the phosphate backbone via arginine residues.

We conducted additional CD melting curves with 16 equivalents of RGGRGGRGG added to TERRA RNA, comparing the thermodynamic stabilization to the native peptide RGGFGGRGG (ΔT_m_ = 9.3°C). Our study showed that upon the addition of 16 equivalents of the mutated peptide RGGRGGRGG, there was no stabilization of the TERRA rG4 structure; instead, a minor destabilization was observed (Supplementary Fig. S13A). Compared to the native peptide, the lack of stabilizing effect suggests that although the mutated RGGRGGRGG peptide may still interact with the TERRA rG4 structure, the phenylalanine residue is essential for π–π stacking within the guanine tetrad, providing structural stabilization.

We tested an additional non-interacting peptide sequence (LGKGGYGKV) retaining 2^+^ charge. This peptide substitutes the positively charged arginine for lysine, the aromatic phenylalanine for tyrosine, and disrupts the canonical RGG RNA-binding motif. LGKGGYGKV had no stabilizing or destabilizing effect on the melting point or folding capabilities of the rG4 structure, suggesting a complete lack of interaction (Supplementary Fig. S13B). This further suggests that interaction and stabilization of the TERRA rG4 by RGGFGGRGG is not driven exclusively by electrostatics—both the arginine and phenylalanine residues, as well as the RGG motif, are essential.

### Structural model of RGGFGGRGG binding to TERRA rG4

Our mutation studies identified uracil loop residues in TERRA, and phenylalanine and arginine residues in RGGFGGRGG as essential for stabilization of the TERRA rG4 structure. To confirm the significance of these components and to model a mechanism of interaction, we employed multidimensional NMR spectroscopy to probe through-space interactions between RGGFGGRGG and TERRA in the presence of potassium ions [93].

**Figure 5.**
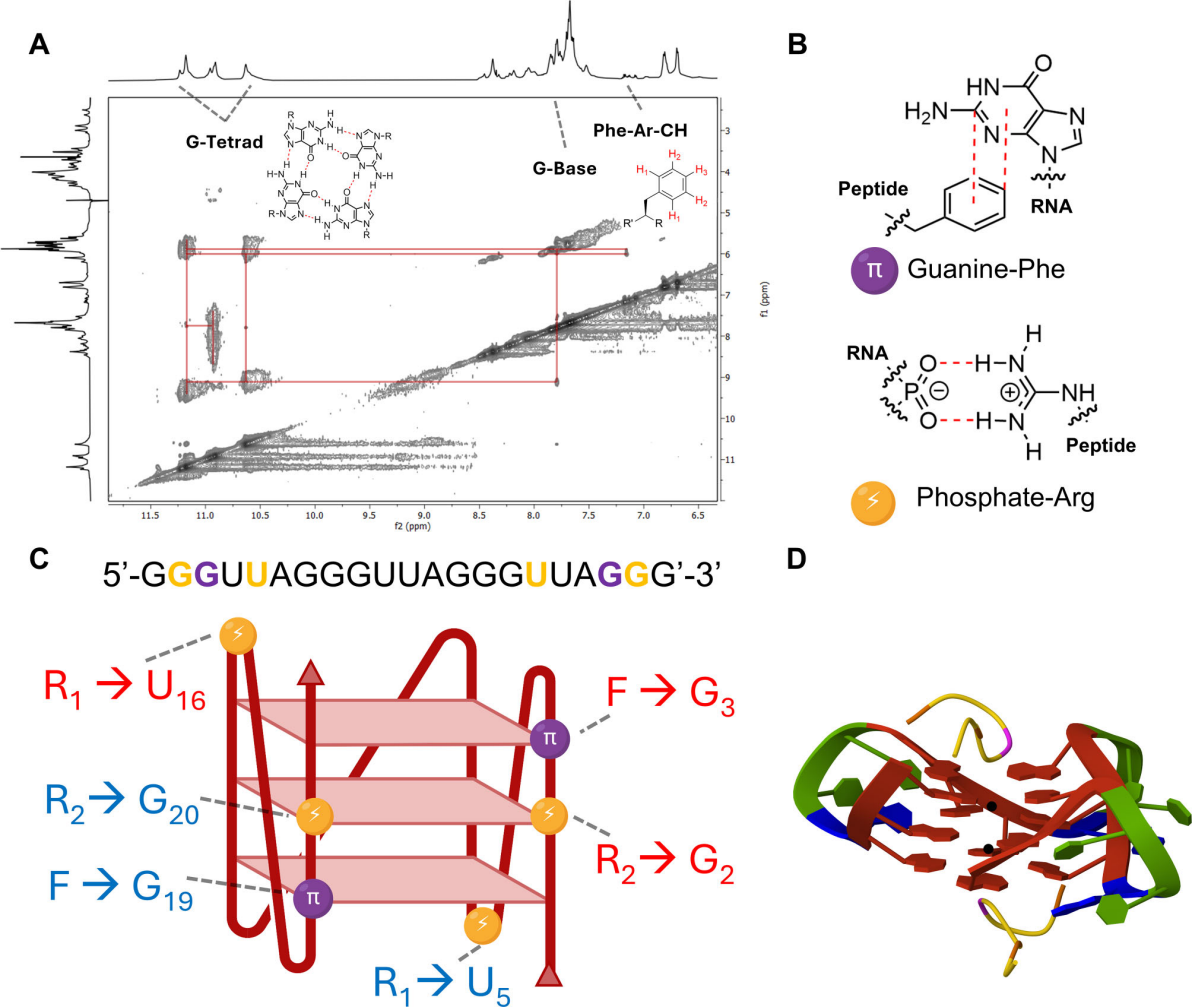
Proposed binding interactions between the RGGFGGRGG peptide and TERRA rG4 in the presence of potassium. (**A**) ^1^H-^1^H NOESY NMR data showing crosspeaks between phenylalanine aromatic protons of RGGFGGRGG and the guanine-tetrad base protons of the TERRA rG4. (**B**) Molecular representation of phosphate-arginine (guanidinium) and guanine-phenylalanine interactions present in the host-guest complex. (**C**) Schematic representation of the TERRA rG4 structure, showing proposed interaction locations as derived from 2D NMR spectroscopy. Yellow dots represent proposed phosphate-guanidinium interactions. Purple dots represent proposed guanine-phenylalanine π-stacking. Interaction locations between the RNA and peptide phenylalanine (F) and arginine (R) are colour-coded for the two binding events. (**D**) AlphaFold 3 computational prediction validating our proposed model. Two RGGFGGRGG peptides bind symmetrically to the top and bottom of the TERRA rG4 structure. The guanine tetrad (red) is being templated by potassium ions (black). Phenylalanine (pink) is π-stacking with guanine, and arginine (orange) is interacting with the phosphate backbone associated with one guanine and one uracil (blue). Adenine (green) does not have a detected interaction with the peptide. Created in BioRender. Cox, L. (2025) https://BioRender.com/d11mn99.

Using ^1^H-^1^H Nuclear Overhauser Effect (NOESY) NMR spectroscopy, we observed strong NOE crosspeaks between the phenylalanine aromatic protons (δ 7.2 ppm) and the guanine tetrad imino-exchange protons (δ 10.5–11.5 ppm, Fig. 5A), as well as additional crosspeaks with guanine base protons (δ 7.6 ppm, Supplementary Fig. S14). These crosspeaks suggest the phenylalanine is π–π stacking with the guanine tetrad on one extremity of the rG4 structure. Additional NOEs were observed between the phenylalanine β-CH_2_ protons (δ 2.5–2.7 ppm), guanine sugar protons (δ 5.8 ppm), and guanine base protons (δ 7.6 ppm), providing further support for an interaction between phenylalanine and guanine, as our mutation studies suggested (Supplementary Fig. S14).

We also observed NOE crosspeaks between β-CH_2_ protons of arginine (δ 1.7 ppm) and both the 2′/3′ ribose protons associated with one uracil and one guanine (δ 3.0–4.5 ppm, Supplementary Fig. S15), suggesting guanidinium-phosphate hydrogen bonding and electrostatic interactions on the external phosphate backbone of the G4 structure [74]. The precise assignment of these protons in the RNA sequence is moderately ambiguous, as it is not possible to determine which specific uracil/guanine is interacting, as we cannot resolve individual bases without isotope labelling [95]. Saturation transfer difference (STD) NMR spectroscopy epitope mapping mirrored NOESY structural data, showing strong proximities between the peptide with guanine bases and guanine/uracil sugars (Supplementary Fig. S16).

Combining the mutation studies and NMR data with our 2:1 peptide-to-RNA symmetrical stoichiometry from NMR titrations, we propose a model where the peptide binds symmetrically to both the top and bottom of the G4 structure. In both binding events, we predict three interaction sites: two arginine residues forming electrostatic and hydrogen bonds with the TERRA phosphate backbone associated with one guanine and one uracil (Fig. 5B, bottom) [74], and one phenylalanine π–π stacking with the guanine tetrad (Fig. 5B, top).

To validate this model, we generated a computational prediction using AlphaFold 3 [95]. We first modelled the native TERRA sequence with 2 potassium ions, which produced a correctly folded parallel rG4, validating AlphaFold 3 as capable of predicting non-canonical RNA structures. After, we added two copies of the RGGFGGRGG peptide, and the proposed model agreed with our mechanism following simulation (Fig. 5D). The model places the peptides symmetrically at the top and bottom of the G4 structure, with the same three non-covalent interactions between each peptide and the RNA. In the AlphaFold model, phenylalanine (F_4_) π stacks with guanine (1F_4_ → G_3_, 2F_4_ → G_19_), the first arginine (R_1_) in each peptide interacts with the phosphate backbone associated with uracil (1R_1_ → U_5_, 2R_1_ → U_16_), and the second arginine (R_7_) in each peptide interacts with the phosphate backbone associated with guanine (1R_7_ → G_2_, 2R_7_ → G_20_).

Together, the experimental and computational data combined provides strong reasoning for the proposed interaction mechanism for the minimal peptide RGGFGGRGG interacting with a short model of human telomeric RNA. This mechanism provides insight into the harmonic cooperation between aromatic and cationic residues to stabilize the TERRA rG4 structure through a combination of π–π stacking and guanidinium-mediated electrostatic templation.

## Discussion

RNA G-quadruplex (rG4) structures in long non-coding RNAs (lncRNAs) are emerging as areas of therapeutic interest [96]. Their structures *in vivo* are shaped by not only physicochemical conditions—ionic strength, monovalent cation concentration, and pH—but also the macromolecular environment, including interactions with RNA binding proteins (RBPs) [55, 56, 58, 97] or other nucleic acids [98] and macromolecular crowding [59], features that are often overexpressed in proteins containing IDRs, which typically bind RNA structures [18–20, 35–37].

Currently available G4 stabilizers are limited to small molecule ligands—TMPyP4, BRACO-19, PhenDC3 & PDS [99–102]. While these ligands bind to and stabilize G4 structures with high affinity, they often show limited discrimination between DNA and RNA G4s [101]. Furthermore, they tend to bind and/or stabilize a diverse range of G4 structures with little to no sequence selectivity and can also bind other nucleic acid secondary structures (e.g. i-motifs, triplexes, and even duplex or single-stranded regions) [82, 103]. This promiscuity lacks the discrimination and biocompatibility required in cellular applications.

G4 structures are stabilized through two main mechanisms: *endo*- and *exo*-stabilization. Monovalent cations stabilize G4s via *endo*-stabilization, coordinating with the central guanine tetrad [9] and promoting Hoogsteen hydrogen bonding. Small molecule stabilizers (e.g. TMPyP4) are thought to work through both *endo-* and *exo-*stabilization via intercalation and end-stacking terminal tetrads, impeding unfolding [99]. Given our NMR data, we propose that the stabilization of rG4s by RGGFGGRGG relies on *exo*-stabilization through interactions with the external phosphate backbone of the RNA structure and π–π stacking with the terminal tetrads. These external interactions are the main mechanism for cellular protein stabilization of rG4s in biological systems [29, 55, 86–88].

Peptide-based targeting agents and therapeutics offer orthogonal interactions, leading to inherent specificity and the ability to rationally design sequences to form transient, tunable interactions with biomolecules, while being extremely viable for scalable synthesis and remaining biocompatible. Clinically validated peptide-based drugs (e.g. semaglutide) provide examples of macromolecular interactions being used with therapeutic and commercial success [104]. The RGGFGGRGG peptide demonstrates that even a minimalistic 9-mer peptide can provide selective recognition of complex RNA structures *in vitro* while retaining biological function, using only a small set of highly conserved amino acid residues [105]. This minimalistic design could be expanded into a library of short RGG-based peptides by systematically varying glycine content to modulate structural flexibility and performing orthogonal screens to probe, stabilize, or inhibit lncRNA secondary structures. Further, alternate peptide sequences can be rationally derived from IDRs of RBPs such as FMRP, hnRNP A1, FUS, or TDP-43 [55, 106–108], to target their native RNA substrates and mimic functions such as structural stabilization, oncogene regulation, and transcriptional/translational regulation.

While the RGGFGGRGG peptide is a promising minimalist model for selective TERRA rG4 stabilization, there are several limitations to be considered before this approach can be extended to biological systems. The relatively modest binding affinity requires a high stoichiometric ratio of peptide to reach saturation, which is a problem *in vivo* due to low RNA concentration and high competition with RBPs [41]. The flexibility afforded by the glycines is useful as a screening tool to maximize the chance of an interaction, but it reduces the overall binding strength of the peptide. Additionally, while the short length of the peptide is a significant advantage in supramolecular and physical chemistry applications, it may undergo rapid enzymatic degradation *in vivo* [109]. These drawbacks can be partially mitigated by reducing the affinity of cellular proteases to the peptide by making unnatural modifications to the sequence, such as arginine methylation or N/C terminal capping, which can slow degradation [110, 111]. Future advances in delivery devices and targeting strategies may expand the uses of minimalist peptides to compete in the cellular environment.

In summary, this study presents the RGGFGGRGG peptide, derived from the IDR of the RNA-binding protein nucleolin, as a minimalist molecular chaperone that is selective for the human telomeric repeat-containing lncRNA (TERRA) rG4 structure. This peptide works in the absence of monovalent cations and displays selectivity for nucleic acid identity (RNA over DNA), topology (parallel G4 over antiparallel G4), and specific loop base identity (uracil over adenine). Multidimensional NMR and computational modelling agreed on a symmetrical 2:1 peptide:RNA interaction mechanism, in which phenylalanine π–π stacks with guanine tetrads, while arginine residues engage in electrostatic and hydrogen bonding with the phosphate backbone near uracil and guanine residues [53–55, 62]. Together, these conclusions rationalize our observed selectivity of RGGFGGRGG for TERRA, offering a stepping stone for mimicking RBP functions on lncRNA regulation.

## Supplementary Material

gkaf1471_Supplemental_File

## Data Availability

This research did not generate any sensitive datasets. Unaltered raw data are available either in the Supplementary data or upon request. NMR titration data is available via GitHub, https://github.com/pallithordarson/RNA_quadruplex_data, and a Figshare repository, https://doi.org/10.6084/m9.figshare.30453494.
